# Transparent Machine Learning Model to Understand Drug Permeability through the Blood–Brain Barrier

**DOI:** 10.1021/acs.jcim.4c01217

**Published:** 2024-11-19

**Authors:** Hengjian Jia, Gabriele C. Sosso

**Affiliations:** Department of Chemistry, University of Warwick, Coventry CV1 1DT, U.K.

## Abstract

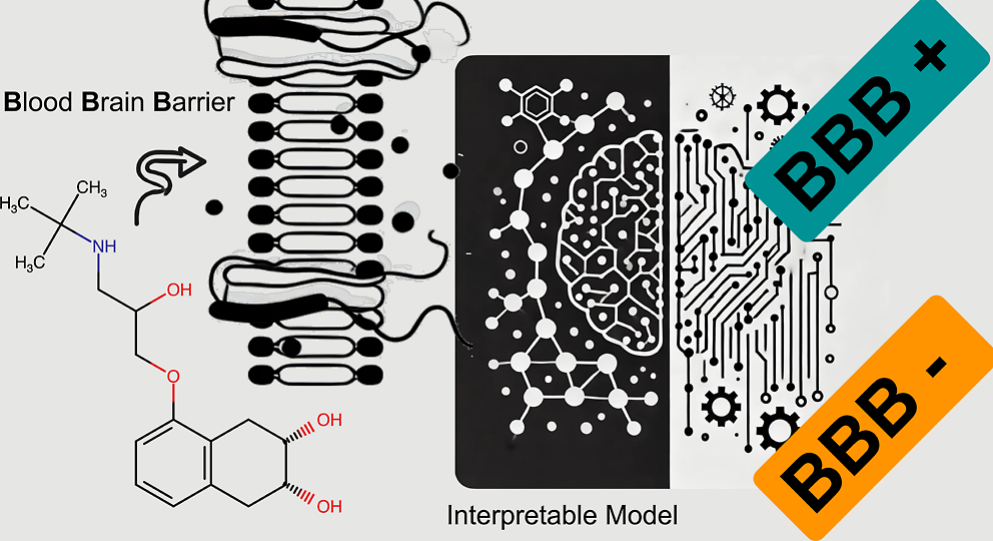

The blood–brain barrier (BBB) selectively regulates the passage of chemical compounds into and out of the central nervous system (CNS). As such, understanding the permeability of drug molecules through the BBB is key to treating neurological diseases and evaluating the response of the CNS to medical treatments. Within the last two decades, a diverse portfolio of machine learning (ML) models have been regularly utilized as a tool to predict, and, to a much lesser extent, understand, several functional properties of medicinal drugs, including their propensity to pass through the BBB. However, the most numerically accurate models to date lack in transparency, as they typically rely on complex blends of different descriptors (or features or fingerprints), many of which are not necessarily interpretable in a straightforward fashion. In fact, the “black-box” nature of these models has prevented us from pinpointing any specific design rule to craft the next generation of pharmaceuticals that need to pass (or not) through the BBB. In this work, we have developed a ML model that leverages an uncomplicated, transparent set of descriptors to predict the permeability of drug molecules through the BBB. In addition to its simplicity, our model achieves comparable results in terms of accuracy compared to state-of-the-art models. Moreover, we use a naive Bayes model as an analytical tool to provide further insights into the structure–function relation that underpins the capacity of a given drug molecule to pass through the BBB. Although our results are computational rather than experimental, we have identified several molecular fragments and functional groups that may significantly impact a drug’s likelihood of permeating the BBB. This work provides a unique angle to the BBB problem and lays the foundations for future work aimed at leveraging additional transparent descriptors, potentially obtained via bespoke molecular dynamics simulations.

## Introduction

The blood–brain barrier (BBB) is a specialized membrane, tightly controlling the movement of chemical compounds between the bloodstream and brain tissue.^1^ It also serves as a protective barrier, preventing foreign substances and potential toxins from affecting brain function and maintaining brain homeostasis.^2^ Thus, the ability to predict the BBB permeability (BBBP) to specific drug-like molecules is crucial to the development of medical treatments targeting the central nervous system (CNS).^2−5^ The development of drugs targeting the CNS has gained increasing importance in recent years.^6^ This is partially due to a rise in neurodegenerative diseases such as Alzheimer’s and dementia, chiefly because of an aging population.^3^ These diseases are playing their part in fueling an increasing burden on healthcare systems.^3,7^

Various in vivo and in vitro studies on drug molecules permeability through the BBB have been conducted.^8−10^ In addition, there has also been research in using molecular dynamics (MD) simulations to predict BBBP.^11,12^ While in vivo and in vitro methods can be very effective, they are also labor-intensive.^13,14^ MD-driven approaches are typically very computationally intensive.^11,15^

As more research has been conducted in the area, the outcomes have been collated together into large data sets which have subsequently been used to build predictive models of BBBP. For instance, Faramarzi et al.^16^ have constructed a model which correlates 386 features with BBBP. These features have been generated using the Leadscope Enterprise and CASE Ultra software^17,18^ on a data set of 921 compounds. The most notable of recent data sets include the MoleculeNet BBBP data set,^19^ which includes 2050 molecules—of which 1567 can permeate the BBB (BBBP+) and 483 cannot (BBBP−), and the more recent B3DB data set,^20^ which contains 7807 molecules—of which 4856 are BBBP+ and 2851 are BBBP–.

Several recent studies have exploited these data sets to build machine learning (ML) models for predicting BBBP. For instance, Kumar et al.^21^ developed the DeepPred BBB model, which uses a convolutional neural network to predict BBBP.^21,22^ Shaker et al.^23^ developed instead the LightBBB model, which utilizes the Light Gradient Boosting Machine (lightGBM) algorithm in conjunction with numerous descriptors obtained via the Dragon software package.^23−25^ In addition, Kumar et al.^26^ have built a Linear Discriminant Analysis (LDA) model using descriptors generated by the alvaDesc^26−28^ software package. The LDA model along with the AlvaDesc package utilized a similarity-based classification read-across structure–activity relationship (c-RASAR) approach.^26−28^ As a further example, the Deep-B model by Tang et al.^29^ combines diverse descriptors and employs computer vision and natural language processing-based models to predict BBBP.

Many of these ML models are complex “black box” models leveraging numerous descriptors—thus lacking interpretability and transparency—trained and validated on relatively small data sets.^30^ Some efforts have been made to create interpretable models, such as the ensemble-based model proposed by Yu et al.,^30^ which boils down to six criteria for predicting BBBP. The criteria identified in Yu et al.^30^ are that molecules which permeate the BBB tend to have zero charge, zero acid groups and zero aromatic C–N bonds. They also typically contain at least one nitrogen heterocycle, at least one seven-membered ring, and less than two hydrogen bond donors. Nevertheless, this model uses a relatively small data set with only 940 molecules, which could impact generalizability.

Rosa et al.^13^ have proposed using the LIME^31^ algorithm with neural networks on extended connectivity fingerprints^32^ to attempt to create an explainable model. However, the LIME algorithm itself is a model-agnostic method of attempting to explain ML models. As such, it treats models as a black box algorithm and attempts to explain them through local optimization. In our opinion, this itself is a black box approach to explaining an already black box algorithm and cannot provide a transparent solution.^31^

Wu et al.^33^ have also proposed another method to achieve interpretable ML models using graph neural networks: they^33^ have shown that using their substructure mask explanation method, they can explain graph neural network models on a number of tasks including BBBP. While both their data set and Rosa et al.’s^13^ data set are larger than that of Yu et al.,^30^ they are still small in comparison to commonly used data sets for testing ML models outside the fields of chemistry or pharmacy—such as the MNIST,^34^ ImageNet,^35^ or LAION-5B^36^ data sets. Wu et al.’s^33^ data set contained only 1859 molecules. Rosa et al.^13^ used the MoleculeNet BBBP data set^19^ which only contained 2039 molecules. Wu et al.^33^ have only 426 molecules in their data set being BBB-nonpermeable. The MoleculeNet data set also only has 479 BBB-nonpermeable molecules. Recent research by Kretschmer et al.^37^ highlighted that the choice of data, particularly the coverage of drug-like molecule space, could significantly affect model generalizability. Kretschmer et al.^37^ have demonstrated that many data sets, including the above-mentioned MoleculeNet BBBP data set, may not provide adequate coverage. Consequently, we argue that models trained on these data sets may not effectively generalize to novel drug-like molecules.

In summary, Yu et al.,^30^ Rosa et al.,^13^ and Wu et al.^33^ rely upon quite complex ML methods to produce interpretable predictions. We demonstrate that it is possible to achieve a comparable predictive power using much simpler methods, which are interpretable due to their transparent nature.

Here, we have utilized the clique descriptor we have introduced in Ref (38) to build a transparent ML model trained on the large B3DB data set.^20^ The clique descriptor dissects a given molecular structure into smaller fragments or functional groups, which can then be transformed into a one-hot vector defined by the number of cliques present in each molecule. Using this uncomplicated descriptor, we have built a random forest model and we have trained it on the B3DB data set to predict the BBBP of drug-like molecules. We found that while our descriptor is, in our opinion, relatively simple, our model matches the performance of the much more sophisticated LightBBB and Deep-B models.

Crucially, the nature of the clique descriptor has allowed us to make a direct connection between functional groups present and BBBP. Specifically, we employed a naive Bayes model as an analytical tool to estimate the extent to which the presence of a given clique (i.e., molecular fragments and/or functional groups) correlates to BBBP. While the naive Bayes model does not carry the same predictive power as a random forest and operates with the assumption of conditional independence, it allows us to estimate the probability of permeating through the BBB for each clique being present. In fact, we have found that certain functional groups, such as organofluorines and some ring structures, show a strong positive correlation with BBBP. Some of these structure–function properties are consistent with the existing literature, which gives us confidence in the validity of our methodology. However, we have uncovered some unprecedented correlations between BBBP and the molecular structure, which, to our knowledge, have not been previously reported elsewhere.

In summary, this work puts forward a simple ML model that nonetheless rivals in terms of accuracy the performance of the state-of-the-art, much more sophisticated methodologies. In addition, we have been able to leverage the straightforward nature of our descriptors to shed new light onto the structure–function relation underpinning the tendency of a given drug-like molecule to percolate through the BBB.

The manuscript is organized as follows: we first detail our chosen data set and our application of the clique descriptor; we then describe how we construct our ML model; finally, we will discuss the various insights that the clique descriptor can reveal and how they correlate to knowledge from existing literature in the results section.

## Methods

### Data Set

Previous works on the topic of BBBP prediction have utilized a number of different data sets. Here, we have considered the B3DB data set,^20^ which contains 2851 “BBBP negative” (BBBP−) and 4956 “BBBP positive” (BBBP+) entries, for a total of 7807 drug-like small molecules. This data set is substantially larger than the data sets used in previous works.^19,23^

**Figure 1:**
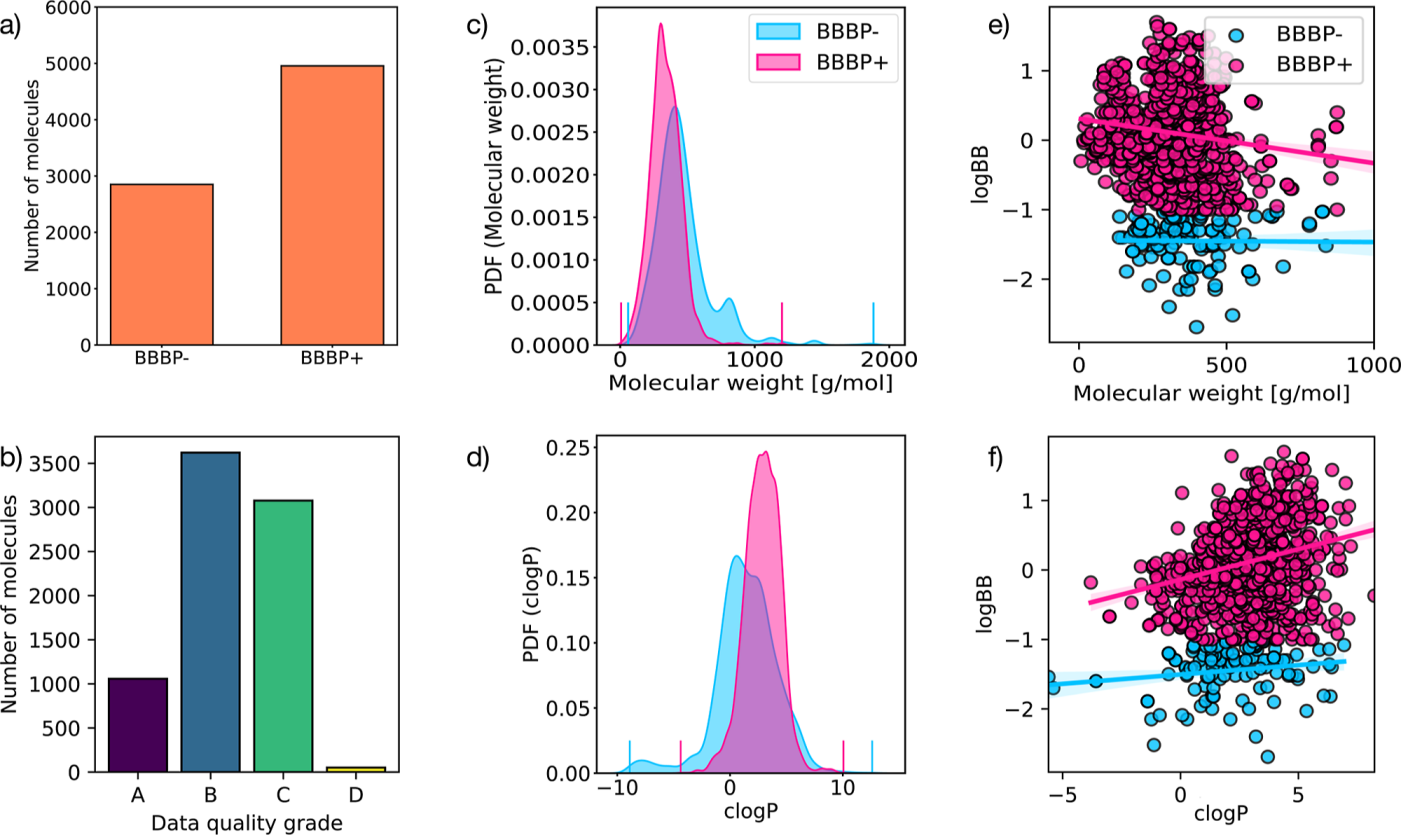
Data set. (a) Histogram representing the occurrence of BBBP–/+ molecules (see text) in the B3DB data set. (b) Histogram illustrating the data quality of the B3DB data set. Grade A is assigned to molecules where a numerical value of log BB is provided. Grade B is assigned to molecules without a reported numerical value of log BB, but all experimental sources classified the molecule as BBBP+/– utilizing a well-defined threshold of log BB = 1. Grade C is assigned to molecules without reported log BB values or classification thresholds, but all sources agree on the BBBP± classification. Grade D is assigned to molecules with conflicting estimates of BBBP ±. (c) Probability density function [via a kernel density estimation, (KDE)] of the molecular weight of each molecule in the B3DB data set. The vertical lines represent the [min,max] range of the sample space. (d) Probability density function (via a KDE) of the *c* log *P* partition coefficient of each molecule in the B3DB data set, computed using RDKit. The vertical lines represent the [min,max] range of the sample space. (e) Scatter plot of the molecular weight against log BB in the B3DB data set, for all the molecules for which log BB information is available. (f) Scatter plot of the *c* log *P* partition coefficient against log BB in the B3DB data set, for all the molecules for which log BB information is available. Note that *c* log *P* measures the log of the ratio of concentrations of a compound across a partition of octanol and water at equilibrium. Hence, *c* log *P* is an intuitive measure of lipophilicity and how well a compound can cross a water–octanol partition.

The B3DB data set^20^ contains the following information. Column 1: molecule name; column 2: SMILES string; column 3: International Chemical Identifier; column 4: whether a molecule can permeate the BBB or not (yes/no binary entry); column 5: the “quality” of each data point (discussed below). The data set also contains a column reporting the log BB for selected compounds. The log BB for a given molecule is defined as
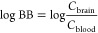
1where *C*_brain_ is the concentration of the molecule found within the brain and *C*_blood_ is the concentration of the molecule within the blood (i.e., outside the brain). The log BB is typically measured once the ratio reaches a steady state.^11,20^ However, this information is only provided for around 1000 molecules of the 7800 molecules present in the data set, which restricts the possibility of building predictive regression models using it. Nonetheless, the log BB information is visualized in Figure 1e,f.

The B3DB data set provides information on the “quality” of each data point.^20^ The B3DB data set is constructed by gathering BBBP results of molecules from a number of different experimental works within the recent literature. In the uncommon cases where different experimental works provide different estimates of the propensity of a given molecule to permeate the BBB, the B3DB data set selects the estimate that occurs most frequently and assigns to that data point a quality “grade” of D. If all recent literature agrees on the BBBP of a certain molecule, but no log BB information is provided, then the data point is assigned a quality grade of C. If there is no log BB information provided, but we do know the threshold the original experimentalist used to convert the log BB value they had (but did not share) to a yes/no for BBBP, then the molecule is assigned as grade B. If log BB information is provided, then the molecule is labeled as grade A. The distribution of these quality grades within the B3DB data set is shown in Figure 1b,^20^ while only 1000 data points carry information in terms of the actual log BB value (A grade). On the other hand, the information relating to classification appears to be rather robust, with only a very minor fraction of the data points being D grade (i.e., data points where the experimental literature provides conflicting estimates of BBBP±).

In order to obtain insights into the overall data set, we have performed some limited exploratory data analysis. Figure 1c shows the distribution of molecular weights in g/mol. We have found that the majority of the molecules in B3DB have molecular weights below 500 g/mol, as is standard for drug-like molecules.^39,40^ However, there are a significant number of other molecules with greater molecular weights.

We also display the *c* log *P* partition coefficient, computed via RDKit, of the molecules in the data set. The *c* log *P* partition coefficient measures the log ratio of the concentration of a molecule across a water–octanol partition. Thus, it is an intuitive measure of lipophilicity that quantifies the extent to which a given molecule can cross a hydrophilic–hydrophobic partition. For the majority of our molecules, both BBB-permeable and BBB-nonpermeable have a *c* log *P* <5, as shown in Figure 1d. This is also commonly observed for drug-like molecules,^40^ but there does appear to be a significant number of molecules with greater *c* log *P*. Based on Lipinski’s rule of five,^40^ this analysis suggests that the B3DB data set includes both drug-like molecules as well as a small but significant number of non-drug-like molecules. In addition to this, we also notice (see Figure 1c,d) that the BBB-permeable molecules in the B3DB data set seem to have a marginally higher *c* log *P* on average and a marginally lower molecular weight. This aligns with existing literature which suggests that molecules which permeate the BBB tend to be small and lipophilic.^41^

We further explored this by visually examining the relationship between log BB and *c* log *P* coefficients. Since B3DB uses −1 as the threshold, all molecules with log BB ≥ −1 are regarded as BBBP+. In Figure 1e,f, we investigate the correlation between *c* log *P* and molecular weight with log BB, respectively. We observe that the log BB information present in the B3DB data set supports the existing literature in that molecules which permeate the BBB tend to be small and lipophilic. We report the Pearson correlation coefficient (PCC) between MW and BBBP– or BBBP+, as well as the PCC between *c* log *P* and BBBP– or BBBP+ in Table 1. We split this table by BBBP– molecules and BBBP + molecules. Note that for BBBP– molecules, log BB has weaker correlation with molecular weight and *c* log *P*. However, for BBBP+ molecules, log BB has stronger correlation with molecular weight and *c* log *P*. The correlation is also shown visually in Figure 1e,f.

**Table 1:** Correlation between Molecular Weight or *c* log *P* and BBBP–/+ Classes^a^

feature	BBBP–	BBBP+
molecular weight	–0.017	–0.169
*c* log *P*	0.177	0.260

a PCC between molecular weight or *c* log *P* and BBBP–/+ classes.

### Descriptors

In cheminformatics, one of the key questions is how to translate a molecular structure into a set of mathematical objects (often labeled as “descriptors” or “features” or “fingerprints”) that can be fed into the ML algorithm of choice. Selecting these descriptors is far from being a trivial process: while utilizing combinations of multiple (up to thousands) different descriptors might lead in some cases to superior numerical accuracy,^41^ this approach often results into an entirely noninterpretable model. This is a major drawback for scientists, as these models do not provide us with any insight with respect to the structure–function relationship underpinning their properties.^30,41^ In fact, we have recently argued^38^ that utilizing a small set of interpretable or “transparent” descriptors might be beneficial in those cases where specific insights into the origins of the functional properties of a given drug molecule are needed.

**Figure 2:**
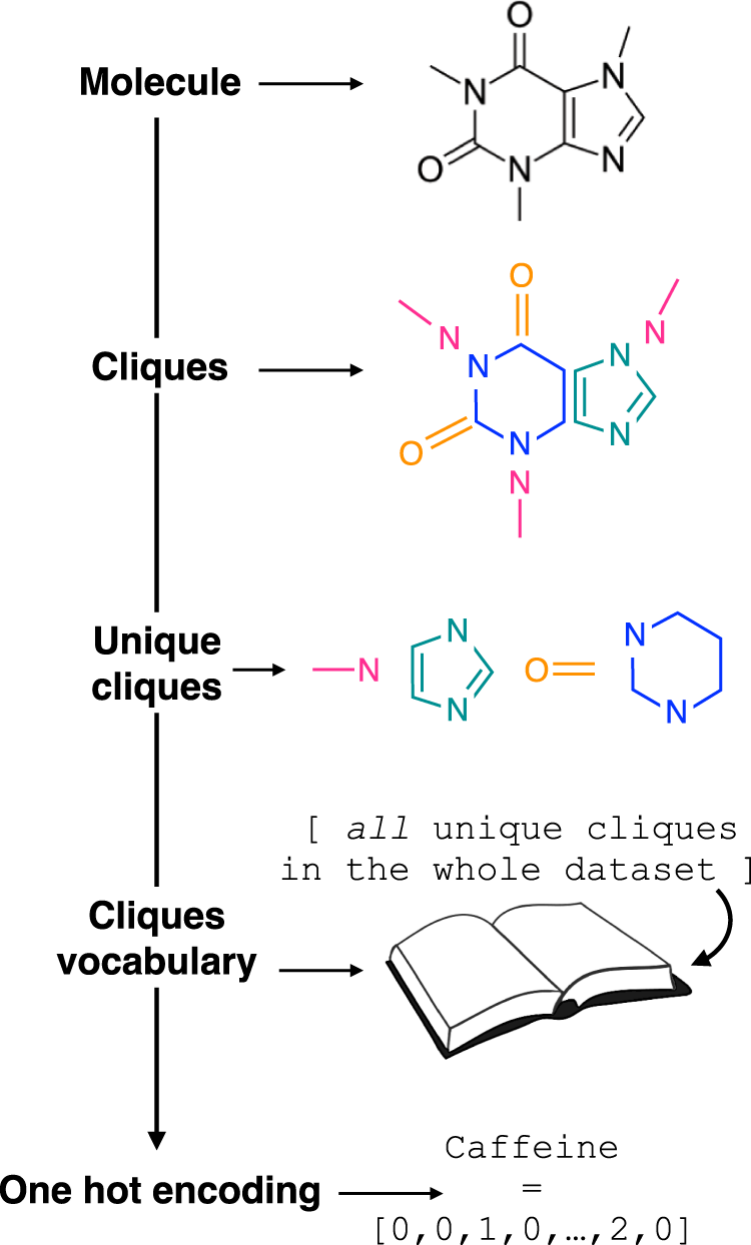
Clique descriptor. Schematics illustrating the idea behind the implementation of the clique descriptor. Adapted from Bernard et al.^38^

Following this strategy, here we have elected to utilize the so-called “clique” descriptor introduced by Barnard et al.^38^ This descriptor leverages simple concepts from natural language processing and consists of breaking down each molecule into a set of functional groups and/or molecular fragments—which we label as “cliques”. These cliques are then used to build a “vocabulary” containing all the unique functional groups and/or molecular fragments present within the data set. From this, each clique can be encoded via a one-hot vector,^38^ and each molecule can be represented by summing the one-hot vectors of all the cliques present within the molecule. This process is summarized in Figure 2. The clique descriptor is thus transparent in nature, as it contains specific, accessible information with respect to each functional group and/or molecular fragment within the molecules. This is in contrast to the extended connectivity fingerprints^32^ used by Rosa et al.,^13^ which—in our personal opinion—lead to both more complex and less transparent descriptors.

We note that, given the limited amount of information contained within the clique descriptor, we have chosen to not perform any feature selection in the context of our random forest models. In addition, the naive Bayes approach that we have also used can be considered as a form of feature selection in itself.

It is worth emphasizing that this descriptor does not contain any information about the connectivity of the different fragments, meaning that it encodes very little information about the structure of a given molecule as a whole. It excels, however, in highlighting the “chemistry” of a given molecule by effectively providing a coarse-grained representation of its functional groups. As such, the clique descriptor leads to accurate predictions in scenarios where the dominant degrees of freedom are the chemical properties of the drugs, as opposed to their three-dimensional structure.

The relative abundance of each clique within the data set can be accessed via the https://github.com/gcsosso/BBBP_CLQ.git repository, which contains all the data relative to this work. The data sets we used are also available at https://zenodo.org/records/13788928.

### Random Forest

A random forest^42^ is created by growing a number of decision trees on a random subset of the data. When predicting results for new data, each tree makes a prediction independently, and the overall result is obtained by the decision trees voting on their predicted class. In this work, the clique descriptors are fed into a random forest with 64 decision trees to predict BBBP. We grow each tree in the random forest by minimizing the Gini impurity metric at each node of the tree.^43^ For each node of the tree, the Gini impurity metric is defined as
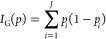
2where *J* is the total number of classes, *p* is the vector of proportions for all classes present at the node, and hence, *p*_*i*_ is the proportion of data points belonging to class *i* at the node. Intuitively, the Gini impurity seeks to maximize the predictive power of each branch of the decision tree.

We ultimately chose the random forest as our choice of ML model as it is relatively quick and simple to construct while still being a very powerful classifier. However, we note that we do not expect the choice of this specific algorithm to bear any impact on the predictive capabilities of our model, chiefly due to the simplicity of the clique descriptors we are utilizing.

In order to deploy a random forest, we use the standard scikit-learn package.^44^ To keep our methodology simple and avoid overfitting hyperparameters, we used the default hyperparameters from the scikit-learn package. We did not perform any hyperparameter tuning, as we found the default hyperparameters to work well. In fact, we noticed that the performance of our random forests was largely unaffected by the number of trees. The results reported in this work have been obtained by utilizing 64 trees.

To validate our results, we use repeated *K* fold cross validation with 5-fold and 10 repeats. This is done by randomly dividing the data set into 5 equally size subsets. We then select one of the five subsets as the validation set and train on the rest. We repeat for all 5 possible validation subsets and then repeat the whole process 10 times with completely new splits each time. Specifically, we do this using the scikit-learn *K*Fold function. We ensure that each repeat is statistically independent by passing it a new random seed each time. Our final results are then obtained by averaging across all repeats and all folds.

We quantified the performance of our models via a diverse portfolio of metrics, namely, the Matthews correlation coefficient (MCC),^45^ the area under the receiver operating curve (AUC) (which is especially suitable for classification problems involving imbalanced data sets), and the sensitivity (SN) and the specificity (SP) for the BBBP+ class. SN measures how well our model performs at detecting BBB-permeable compounds, while SP measures how well our model performs at detecting BBB-nonpermeable compounds. The choice of these metrics will allow us to compare our results to the existing literature on the subject. Note that for all of these metrics, higher values are indicative of a better performance of the model. In particular, a perfect classification algorithm would have a score of 1.0 in all of these metrics, while a set of entirely random predictions would score a value of zero for SN, SP, and MCC and an AUC of 0.5.

### SMOTE

As illustrated in Figure 1, the B3DB data set is significantly skewed toward BBBP+ compounds. As such, we have applied an adapted version of the conventional synthetic minority oversampling technique (SMOTE^46^) to the data. This is a useful strategy, as some classifiers—including random forests—can often struggle with skewed data sets.^47,48^

The SMOTE algorithm works by first sampling two random data points from the minority class and then drawing a straight line between the two random points. A new feature can then be generated by sampling from this line with a uniform probability. In other words, SMOTE works by linearly interpolating the space between the minority class and sampling from it. SMOTE repeats this method until there are an equal number of data points in both classes.

However, the SMOTE algorithm was originally designed for continuous data. As the space of our descriptor features is built by summing one-hot vectors of present cliques, it is therefore an ordered discrete value. As a result, we can round the result of SMOTE in order to obtain a suitable synthetic feature. This maintains the randomness of SMOTE while sensibly discretizing the SMOTE algorithm for our specific needs.

It should be noted that SMOTE does not generate new information; it simply interpolates data points within the existing data set. Additionally, SMOTE is applied exclusively to the training set and is not used on any of the validation sets.

### Naive Bayes

In addition to using a random forest to try to predict BBBP, we also employ the naive Bayes model as an analytical tool. In particular, we use naive Bayes in order to build a structure–function relationship between a given molecule and its propensity to permeate the BBB.

To do this, we first transform the clique descriptor into a binary descriptor. Instead of looking at how many cliques of a given type are present within a given molecule, we only concern ourselves with whether it is present or not. A standard Bernoulli naive Bayes classifier can then be built.^49−52^ In particular, the Bernoulli naive Bayes algorithm attempts to estimate
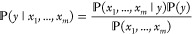
3where *y* is the Bernoulli random variable indicating whether a molecule can permeate through the BBB and *x*_*i*_ is the Bernoulli random variable indicating whether the *i*th clique is present in that particular molecule. Applying the naive Bayes assumption of conditional independence
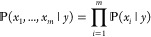
4we obtain
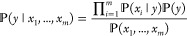
5

While the normalization factor  can in principle be computed, it is often disregarded in practice, as it does not influence the decision-making process of the naive Bayes algorithm. The same factor does not depend on *y*, which in turn yields
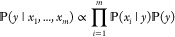
6i.e., the standard Bernoulli naive Bayes estimator. However, we sought to obtain an estimate for *P*(*y*|*x*_*i*_). Since each clique has different marginal likelihoods of occurring, we must compute  to estimate this. Fortunately, since we are working with binary variables, each  is tractable and can be calculated as . Therefore, we obtain
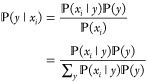
7which is the marginal probability of permeating the BBB given the presence of the *i*th clique. This therefore creates a probabilistic metric for estimating the extent to which each individual clique contributes to BBBP.

It is worth noting that other methods of analyzing our descriptors exist, such as the LIME^31^ algorithm deployed by Rosa et al.^13^ However, the naive Bayes approach is far simpler and also has no hyperparameters to tune, making it far easier to deploy and to understand. Naive Bayes is also a deterministic and nonparametric model, so there are no parameters to optimize either. However, the trade-off is that naive Bayes is certainly not a model and descriptor agnostic approach unlike LIME, as naive Bayes itself is a model.

## Results and Discussion

### Model Performance

We have summarized the performance of our models in Table 2. We obtained our results by averaging over ten statistically independent sets of 5-fold cross validation. Our results were measured using SN, SP, and MCC, as well as AUC.^45^ The SN measures our ability to detect BBBP+ compounds by dividing the true positives by all positives. Likewise, the SP measures our ability to detect BBBP– compounds. Both MCC and AUC incorporate SN and SP in order to give an overall picture of the performance of the model at detecting both BBBP+ and BBBP– compounds. We noticed that SMOTE substantially increased the specificity of the model (84%, to be compared with 78% without using SMOTE) at the cost of sacrificing some of its sensitivity (90% versus 94% without SMOTE), see Table 2.

**Table 2:** Comparison of Our Models with the State of the Art^a^

model	SN (%)	SP (%)	MCC	AUC
LightBBB	86	55	0.43	
Deep-B	85	64	0.49	0.83
Deep-B (without data augmentations)	**95**	41	0.41	0.80
LDA			0.6	0.92
cliques	90	**84**	**0.74**	**0.95**
cliques (without SMOTE)	94	78	0.74	**0.95**

a We report the SN, the SP, the MCC, and the AUC for our models (cliques, with or without the usage of the SMOTE algorithm). Note that we do not use apply SMOTE to any classification sets and only the training set when it is used. We also display the performance of recent models trained on the same B3DB data set (see text). Specifically, LightBBB refers to the work of Shaker et al.,^23^ while Deep-B refers to the work of Tang et al.^29^ The latter utilizes two variants, one with and one without their form of data augmentation. In addition, LDA refers to the Linear Discriminant Analysis model by Kumar et al.^26^

The results for Deep-B and LightBBB were obtained using an approximately 60/40 train/test split with no cross validation.^29^ The results for LDA were obtained using an approximately 1:1 train/validation split.^26^ Kumar et al.^26^ also performed 20 statistically independent sets of 5-fold cross validation. We have chosen not to include the results obtained by Faramarzi et al.^16^ as they have utilized a much smaller data set.

Note that utilizing the SMOTE algorithm resulted in zero net change for both the MCC and AUC metrics. This is expected to a certain extent, as SMOTE does not actually produce any real new data for the minority class; it merely interpolates linearly between data points in the minority class to generate synthetic features. This does indicate that attempting to improve specificity through engineering the data itself is not likely to be effective at producing meaningful improvements on overall model performance.

Our models outperform the LightBBB, Deep-B, and LDA models. This is a striking result, as our model utilizes a single class of rather elementary descriptors, i.e., the clique descriptors described in the Methods section, in conjunction with a run-of-the-mill random forest algorithm. In stark contrast, the LightBBB^23^ model uses a gradient-boosting framework (light GBM^24^) in conjunction with a substantial set of different descriptors generated by the Dragon software package.^25^ Similarly, the Deep-B model leverages an elaborate neural network model combined with a diverse array of features, including MACCS^53^ and Morgan^32^ fingerprints.

### Interpreting the Model

**Figure 3:**
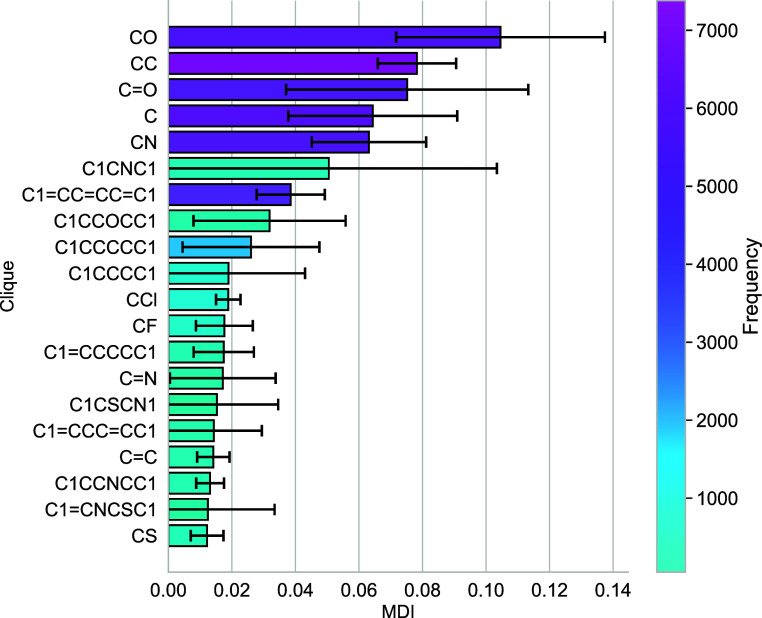
MDI relative to our random forest model, reported for the 20 most significant cliques. The error bars represent the standard deviation of the decrease in impurity for each clique across all the trees present in the random forest. Note that we have ignored any clique which occurs in less than 50 different molecules in the data set.

As our model exclusively employs the clique descriptor, which in turn is transparent, we have the unique opportunity to make a connection between the presence of a given clique within a molecule and the propensity of the molecule to cross the BBB. There exist a number of potential avenues to try to understand the effect of each clique on BBBP. One straightforward approach is to measure the mean decrease in impurity (MDI)^43^ for each clique. The MDI quantifies the extent to which a given feature contributes to reducing the impurity (see the Methods section) in the prediction. The MDI is a non-negative number, which we have normalized so that the sum of the MDI over all the cliques is equal to one: the higher the MDI, the more impactful the feature in question. We report the 20 most significant cliques, according to their MDI, in Figure 3. The complete list is available at https://github.com/gcsosso/BBBP_CLQ.git.

However, the MDI only identifies which cliques are deemed important by the random forest algorithm, without indicating directly whether a clique is likely to promote or hinder the permeability of the BBB. In addition, the MDI for several of the cliques reported in Figure 3 is characterized by a high standard deviation, which limits the significance of this metric. Finally, the MDI does not provide us with an actual probabilistic metric for the BBBP tendency associated with each clique.

To address these limitations, we have computed for each clique in the B3DB data set (via a Bernoulli naive Bayes approach, see the Methods section) P(BBB+), i.e., the probability for a molecule to successfully cross the BBB given the presence of that particular clique. The naive Bayes model is a weaker classifier compared to our random forest model with lower performance (MCC = 0.53 and AUC = 0.87) in predictive tasks. However, we note that (i) the naive Bayes model still outperforms state-of-the-art results (see Table 2) and (ii) the primary goal of using the naive Bayes model is not for accurate predictions but to compute probability statistics on the clique descriptors.

**Figure 4:**
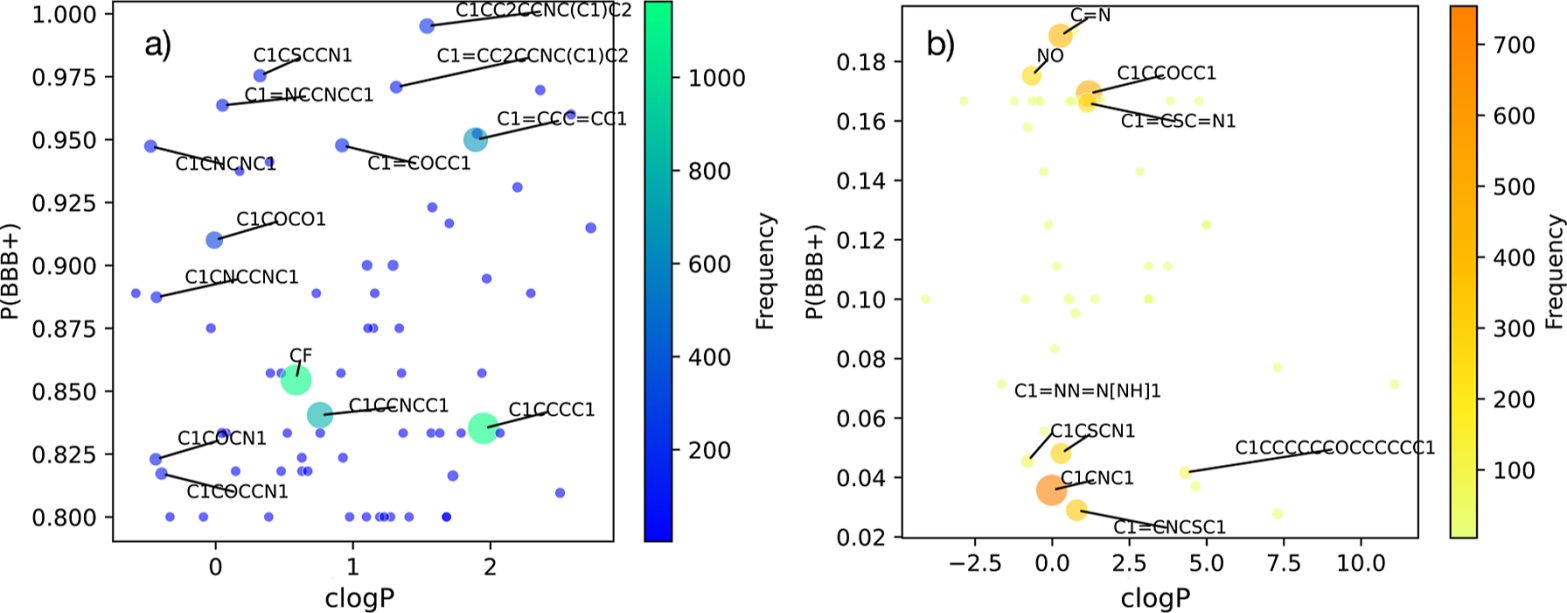
Structure–function relation between the presence of molecular fragments and the probability of a given molecule to cross the BBB. Scatter plot of the probability for a given molecule to successfully cross the BBB, P(BBB+), given the presence of a specific clique, against the *c* log *P* value of that same clique. The size and the color (see color bars) of each point are proportional to the frequency by which a given clique is found within the B3DB data set. We have restricted this visualization to (a) cliques for which P(BBB+) is greater than or equal to 0.8 and (b) cliques for which P(BBB+) is equal to or less than 0.2. In other words, panel (a) focuses on those cliques which are connected to high probability for the whole molecule to cross the BBB, and panel (b) focuses on those cliques whose presence seems to substantially hinder the capacity of a given molecule to cross the BBB. We have labeled with the corresponding SMILES strings those cliques that appear in at least 50 different molecules within the B3DB data set.

We report our results in Figure 4. In particular, we focus in Figure 4a on those cliques for which the corresponding marginal P(BBB+) is greater than or equal to 0.8. This focuses on cliques whose presence seems to favor the ability of a given molecule to cross the BBB. Conversely, in Figure 4b, we focus on those cliques for which the corresponding P(BBB+) is equal to or less than 0.2. This focuses on cliques which seem to hinder the capacity of a molecule to cross the BBB. For these selected cliques, we also report their *c* log *P* value (albeit the value of *c* log *P* for an isolated molecular fragment needs to be considered as a qualitative indicator only of its lipophilicity) and the frequency by which we observe them within the B3DB data set. In particular, we have labeled with their corresponding SMILES string those cliques which are present in at least 50 different molecular structures within the data set. Note that the *c* log *P* of each clique is computed by RDKit.

Interestingly, we note that several (nine) of the 20 most important cliques identified by means of the MDI metric (see Figure 3) also have a significant ability to influence the BBBP of a given drug molecule according to our naive Bayes model (see Figure 4). These cliques are C1CNC1, C1CCOCC1, C1CCCC1, CF, C=N, C1CSCN1, C1=CCC=CC1, C1CCNCC1, and C1=CNCSC1.

**Figure 5:**
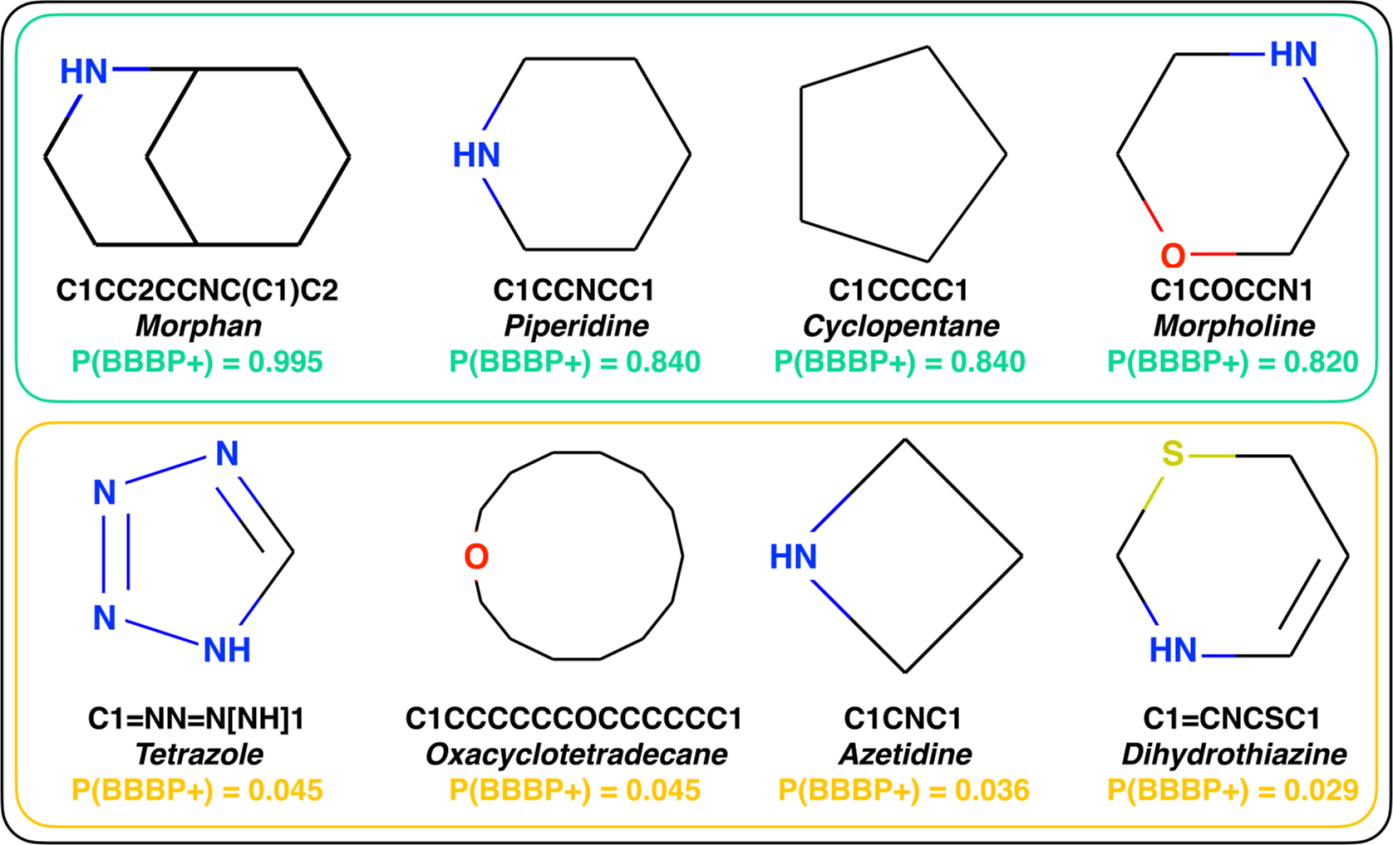
Molecular fragments that have substantial impact on the probability of a given molecule to cross the BBB. We report the molecular structure corresponding to a selection of cliques (see text). The top and bottom rows correspond to cliques associated with very high and very low P(BBB+), respectively.

Additionally, we have found that some of these cliques (for which structures are shown in Figure 5) have been mentioned already in the context of the existing literature about the BBBP. For instance, carbon–fluorine bonds (CF) seem to substantially enhance the ability of a given molecule to cross the BBB. This is corroborated by the work of Sandford,^54^ who found out that the antidepressant drug Prozac owes its ability to permeate the BBB to a fluoromethyl group (a carbon atom connected to 3 fluorine atoms).^54^ Furthermore, Frolov and Vereshchagin^55^ have shown that fluoroethyl groups (2 carbon atoms connected to a fluorine atom) can also enhance the BBBP of drugs—in this case without affecting their function. In addition, another computational study by Faramarzi et al.^16^ also suggests that piperidine is strongly positively correlated with BBBP.

Cyclopentane (C1CCCC1) is also associated with a high value of P(BBBP+). Cyclopentane as a self-standing molecular species is a cycloalkane, which is known to have toxic effects on the brain—in turn, that is suggestive of the capacity of this class of organic molecules to permeate the BBB with ease.^56,57^ We have also found that morpholine (C1COCCN1) has a high probability of permeating the BBB. Though this clique is not very abundant within the B3DB data set, its relevance in the context of the BBBP is supported by the work of Lenci et al.,^58^ which claims that “the presence of a weak basic nitrogen atom and an oxygen atom at the opposite position provides a peculiar p*K*_a_ value and a flexible conformation to the ring, thus allowing it to take part in several lipophilic–hydrophilic interactions and to improve blood solubility and brain permeability of the overall structure”.

Importantly, our analysis enabled us to expand on other existing BBBP literature as well. For instance, Yu et al.^30^ suggest a number of rules for predicting BBBP, one of which is the presence of a nitrogen heterocycle. However, our results seem to be more nuanced in this regard. While we have indeed found that certain nitrogen heterocycles correlate very strongly with P(BBBP+) (such as piperidine, which has a 0.84 marginal probability of permeating the BBB), others show very low marginal probability of permeating the BBB (such as azetidine, with P(BBB+) = 0.04).

From Figure 4, we can argue that the nitric oxide (NO) clique is associated with a low probability of permeating the BBB. While this hypothesis is corroborated by the work of Thiel and Audus,^59^ the same authors suggest that NO can disrupt the function of the BBB itself. In particular, NO can cause structural damage to the BBB and increase its permeability to other compounds. An interesting aspect is that the NO clique does not appear in the B3DB data set as a standalone molecule but only as part of larger compounds. This highlights the ability of our clique descriptor to extract nontrivial insights, despite its simplicity. However, as far as we know, such compounds are still labeled as BBBP– in the B3DB data set, without accounting for their potential to influence BBBP. Further to this, it appears that certain compounds such as tetrazole-based compounds can damage the BBB through inducting production of nitric oxides.^60^ On a similar note is that we predict that tetrazole is associated with low BBBP. This is supported by Danjo et al.,^60^ who showed that pentylenetetrazol is impermeable to the BBB. However, they also demonstrated that pentylenetetrazol can damage the BBB and increase its permeability by inducing the production of excess NO.

Moreover, while we predict that the large ring structure oxacyclotetradecane is likely to be impermeable to the BBB, existing literature suggest this may not hold true under all conditions. According to Drugbank,^61^ erythromycin an antibiotic containing this ring structure is impermeable to the BBB under normal conditions. However, erythromycin can permeate the BBB in meningitis patients, likely because the inflamed tissue is more permeable.

Some of the cliques we have found to be important in terms of influencing P(BBBP+) are not necessarily well-known within the community. As representative examples (also depicted in Figure 5), we highlight morphan and dihydrothiazine, which are characterized by P(BBBP+) values of 0.995 and 0.029.

Finally, we notice in Figure 4 that there does appear to be some correlation between the *c* log *P* of individual cliques (however ill-defined that might be for isolated molecular fragments) and their likelihood of permeating the BBB. Cliques with a high probability of permeating the BBB seem to have a much more limited range of *c* log *P*. This also expands on existing literature, in that both Carpenter et al.^11^ and Geldenhuys et al.^62^ suggest that the *c* log *P* value of the drug molecule as a whole is a significant predictor of BBBP.

## Conclusions

Being able to accurately predict the BBBP of molecules is a vital part of developing pharmaceutical drugs that target the CNS. Within the past few years, several ML-based models have been used to build predictive frameworks capable of assessing the BBBP of small drug-like molecules.

However, the vast majority of these models suffer from a lack of interpretability, thus preventing us from leveraging the outcomes of these computational frameworks to inform drug design and discovery.

In this work, we have used a single, straightforward, and transparent descriptor (the “cliques” descriptor) to build a ML model that, despite its simplicity, outperforms state-of-the-art models trained on the same data set. This result demonstrates that in some cases, “less is more” and allows us to build a structure–function relationship between the molecular structure and BBBP.

As the clique descriptors have no capacity to represent the actual molecular structure as a whole, we also argue that the propensity to permeate the BBB must be chiefly determined by the “chemistry” of the molecule (i.e., the presence of specific functional groups within it) as opposed to its “structure” (i.e., the exact spatial arrangement of said functional groups). Clearly, this is not the case for properties such as the solubility of drug crystalline formulation, where the three-dimensional structure of the molecule and the solid is bound to have a major impact on this particular functional property. However, we can speculate that other properties of relevance for the pharmaceutical industry, such as the toxicity of a given drug-like molecule, might be influenced by “chemistry” to a much greater extent than by “structure”. We plan to investigate this hypothesis in future work.

Our work builds upon the work of others in the field such as Rosa et al.^13^ who seek to highlight the relationship between molecular fragments and BBBP. In particular, we have adopted a simple naive Bayes framework to estimate the probability for a molecule containing a given clique to effectively cross the BBB. This analysis yields some results that are supported by independent investigations within the recent literature, giving us confidence in the robustness of our approach. Moreover, the same analysis gave us well-quantified probabilistic insight into the impact of several molecular fragments on BBBP, thus opening new avenues in terms of the rational design of pharmaceutical drugs that need to cross the BBB.

While the data set we have been using is limited to around 7800 data points, we argue that our model should be fairly transferable to a diverse portfolio of molecular compounds, albeit the reliability of this hypothesis needs to be investigated in future work.

The rather unexpected accuracy of the clique descriptor in predicting the BBBP is, we argue, a byproduct of the fact that the presence of specific functional groups and/or molecular fragments (the “chemistry” of a molecule) within a given drug molecule is much more impactful than the exact three-dimensional molecular structure of the molecule (the “structure” of a molecule) as a whole. The fact that, as far as the BBBP is concerned, chemistry outweighs structure suggests the possibility that other functional properties of relevance in the context of drug design and discovery, such as toxicity or human hepatocyte intrinsic clearance, could also be modeled quite accurately by means of simple descriptors such as the cliques.

Clearly, this kind of analysis might massively benefit from additional data points. Nevertheless, even within the limitations imposed by the data set size, it is refreshing to be able to use such straightforward descriptors as the cliques to infer useful trends with respect to the structure–function relation underpinning the ability of a given drug-like molecule to cross the BBB.

While our results are purely computational, we hope they will serve as a valuable guide for future experimental research. Indeed, we believe that our findings might have the potential to assist in better understanding how molecular chemistry influences BBBP and thus aid in the design of new brain-targeting drugs.

Overall, this work challenges the current paradigm of ML for drug design and discovery, which seems to be dominated by models of ever-increasing complexity, often relying on vast arrays of nontransparent descriptors, possibly due to the ease by which the latter are now becoming readily available to the average user.

We hope that this work will lay the foundations for future investigations aimed at incorporating this model with other models targeting different functional properties, in the attempt to build a computational framework that could be of practical utility to pharmaceutical companies in the context of accelerating the pipeline of drug design and discovery.

## Data Availability

All of the data associated with this study as well as the in-house codes and scripts we have used to obtain and analyze our results (including software dependencies) are publicly available via the following GitHub repository: https://github.com/gcsosso/BBBP_CLQ.git. The same repository includes Jupyter Notebooks that can be straightforwardly used to both replicate and visualize all the results reported in this work. In addition, the data sets we used are also available at https://zenodo.org/records/13788928.
